# Trajectory of AKI and hospital mortality among patients with COVID-19

**DOI:** 10.1080/0886022X.2023.2177086

**Published:** 2023-03-06

**Authors:** Seong Geun Kim, Chung Hee Han, Sung Bong Yu, Hyeseung Lee, Soie Kwon, Yerim Kim, Jeonghwan Lee, Dong Ki Kim, Yun Kyu Oh, Chun Soo Lim, Yon Su Kim, Byung Gun Kim, Jung Pyo Lee

**Affiliations:** a Department of Internal Medicine-Nephrology, Seoul National University College of Medicine, Seoul, Republic of Korea; b Department of Internal Medicine, Seoul National University Hospital, Seoul, Republic of Korea; c Department of Obstetrics and Gynecology, Bagaehospital, Pyeongtaek, Gyunggi-Do, Republic of Korea; d Department of Surgery, Bagaehospital, Pyeongtaek, Gyunggi-Do, Republic of Korea; e Department of Internal Medicine-Nephrology, Keimyung University School of Medicine, Daegu, Republic of Korea; f Department of Internal Medicine, Seoul National University Boramae Medical Center, Seoul, Republic of Korea; g Department of Orthopedic Surgery, Bagaehospital, Pyeongtaek, Gyunggi-Do, Republic of Korea

**Keywords:** Acute kidney injury, renal recovery, mortality, COVID-19

## Abstract

**Background:**

Acute kidney injury (AKI) in COVID-19 patients is associated with poor prognosis. Characterization of AKI by timing and trajectory and early prediction of AKI progression is required for better preventive management and the prediction of patient outcomes.

**Methods:**

A total of 858 patients who were hospitalized due to coronavirus disease 2019 (COVID-19) were retrospectively enrolled from December 2020 to August 2021. The occurrence of AKI was evaluated throughout hospitalization. The hazard ratios (HRs) of mortality outcomes according to the trajectory of AKI were measured using Cox regression models after adjustment for multiple variables.

**Results:**

Among 858 patients, 226 (26.3%) presented AKI at admission, and 44 (5.1%) developed AKI during hospitalization. Patients with AKI at admission or hospital-acquired AKI had a higher risk of mortality than those without AKI, with HRs of 9.87 (2.81–34.67) and 13.74 (3.57–52.84), respectively. Of 226 patients with AKI at admission, 104 (46.0%) recovered within 48 hr, 83 (36.7%) had AKI beyond 48 hr and recovered in 7 days, and 39 (17.3%) showed no recovery from AKI on Day 7. Delayed recovery and persistent AKI were significantly associated with an increased risk of mortality, with HRs of 4.39 (1.06–18.24) and 24.33 (7.10–83.36), respectively.

**Conclusions:**

The onset and progression of AKI was significantly associated with in-hospital mortality in patients with COVID-19. A thorough observation of the recovery trajectory of early AKI after infection is necessary.

## Introduction

Coronavirus disease 2019 (COVID-19) is primarily known to affect respiratory systems, however, the kidney has also been recognized as a vulnerable target organ for infection. The reported prevalence of acute kidney injury (AKI) has been shown to be elevated among individuals diagnosed with COVID-19 in comparison to those without the disease [1]. The occurrence of AKI constitutes a hallmark of COVID-19 and represents a substantial risk factor with the potential to induce unfavorable outcomes among hospitalized patients [2–4].

The pathogenesis behind the impact of COVID-19 on kidney injury is understood to be multifaceted. One possible mechanism of kidney involvement could be similar to that observed during severe acute respiratory syndrome coronavirus (SARS-CoV) infection in the past, including cytokine release syndrome, inter-organ communication, and systemic effects [5–7]. The development of AKI is believed to be the result of both local and systemic inflammatory and immune responses, along with endothelial damage and activation of coagulation pathways and the renin-angiotensin system [8,9]. Although direct kidney infection by SARS-CoV-2 is still a matter of debate, a study largely based on autopsies revealed increased tubulointerstitial fibrosis in COVID-19 patients and suggested a direct kidney infection [10].

Most previous studies of the trajectory after AKI in the pre-COVID era show favorable outcomes after renal recovery compared to nonrecovery, including a lower risk of progression to CKD or mortality [11–14]. However, even in the presence of renal recovery, AKI remains associated with long-term mortality [13,15,16]. Characterization of AKI by timing and trajectory and early prediction of AKI progression might help with risk stratification and allow better preventive management, hospital resource allocation, and patient prognostication in this pandemic. Accordingly, we aimed to investigate patient outcomes according to the time and trajectory of AKI following hospitalization with COVID-19 and to identify risk factors associated with impaired renal recovery.

## Method

### Patient and data collection

The study was retrospective in nature and included a cohort of 1402 patients who were hospitalized with COVID-19 at a single secondary hospital from December 2020 to August 2021. Among them, patients who were under 18 years old (*n* = 100) and those who had been on dialysis because of end-stage kidney disease (*n* = 82) were excluded. Baseline information at the time of admission was not available for 362 patients; thus, a total of 858 patients were ultimately analyzed. The study design was approved by the institutional review board of the Seoul National University Boramae Medical Center (no. 20-2021-88) and complied with the Declaration of Helsinki. The requirement for informed consent was waived by the review board.

Baseline information at the time of admission was collected from electronic medical records and included age, sex, clinical symptoms, comorbid conditions, laboratory data, and ICU admission. The laboratory dataset included complete blood counts, liver function tests, renal function tests, inflammatory markers, and cycle threshold (Ct) values of real-time reverse transcription-polymerase chain reaction (RT–PCR) assays, which are a confirmatory test for COVID-19. Estimated glomerular filtration rate (eGFR) was calculated using the Chronic Kidney Disease Epidemiology Collaboration (CKD-EPI) equation. Underlying chronic kidney disease (CKD) was identified using previous medical records, laboratory data, and patient reports. The study outcome was in-hospital mortality until discharge.

### Definitions

The baseline serum creatinine level was defined as a preadmission outpatient creatinine measurement within the 6 months before admission (*n* = 27), and if not available, the minimum serum creatinine value during hospitalization (*n* = 831). According to the timing of AKI, patients were divided into two groups: those with AKI at admission and those with hospital-acquired AKI that developed after hospitalization.

The classification of hospital-acquired AKI was defined according to the Kidney Disease: Improving Global Outcomes (KDIGO) criteria as: Stage 1, an increase in serum creatinine level by 0.3 mg/dL within 48 h or a 1.5–1.9-fold increase from baseline within 7 days; Stage 2, a 2.9-fold increase in serum creatinine level within 7 days; and Stage 3, a 3-fold or greater increase in serum creatinine level within 7 days or initiation of renal replacement therapy.

AKI at admission was defined using the retrospective diagnostic criteria proposed in the previous literature as follows: Stage 1, 0.66–0.49 times the reference value; Stage 2, 0.5–0.32 times the reference value; and Stage 3, ≤0.33 times the reference value [17]. The reference value was the creatinine value at admission. In addition, patients with AKI at admission were classified according to the rate of AKI recovery, which was adjudicated based on the serum creatinine level at 7 days after admission. Early recovery was defined as a period of reversal to the baseline creatinine level within 48 h, and delayed recovery was defined as reversal occurring between 48 h and 7 days. Persistent AKI was defined as no recovery to baseline level within 7 days after admission.

### Statistical analysis

The expressions of categorical and continuous variables were reported as proportions and means with standard deviations, respectively, if they were found to have a normal distribution based on the results of the Kolmogorov–Smirnov test. For non-normally distributed variables, medians with interquartile ranges were used instead. The chi-square test or Fisher’s exact test was applied to compare categorical variables. In the case of continuous variables, the Student’s *t* test or Mann–Whitney *U* test was applied depending on the normality.

Kaplan–Meier survival curves were drawn and compared between groups using the log-rank test. The hazard ratios (HRs) and confidence intervals for hospital mortality were calculated using a Cox proportional hazards regression model in which multiple variables were adjusted. The odds ratios (ORs) and confidence intervals for delayed recovery or persistent AKI in the AKI at admission group were calculated using logistic regression analysis. All statistical analyses were performed using SPSS software (version 27; IBM, Armonk, NY, USA) and R software (version 3.5.1; R core team, Vienna, Austria).

## Results

### Trajectory of AKI

**Figure 1.**
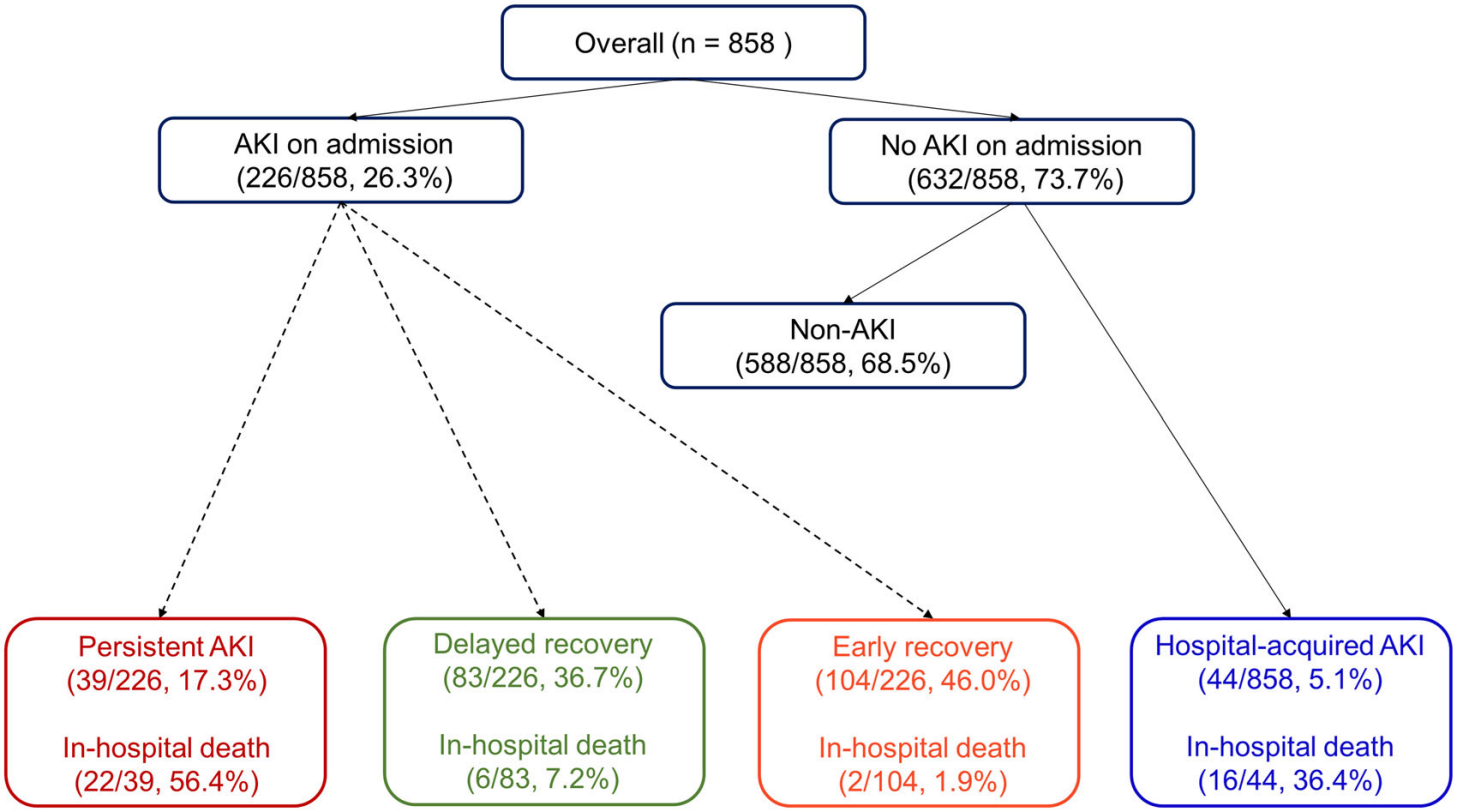
Trajectories of AKI in hospitalized patients with COVID-19.

Figure 1 shows the flow chart of all patients according to the course of AKI. Among 858 patients, AKI was present in 226 (26.3%) patients at the time of admission, and 44 (5.1%) patients developed AKI during hospitalization. We classified the 226 patients with AKI at the time of admission into three groups according to renal recovery. Kidney function recovered to baseline within 48 h in 104 patients, and for 83 patients, recovery took more than 48 h. Thirty-three patients did not recover by the 7th day.

### Patient characteristics by timing of AKI

The mean age of the patients was 56 ± 20 years, and 37.1% of the patients were male. Patients who developed AKI were more likely to be older and have comorbid diseases such as hypertension, diabetes, and CKD. The difference was more pronounced in the hospital-acquired AKI group. Laboratory findings such as hemoglobin, platelet count, serum albumin, C-reactive protein (CRP) and brain natriuretic peptide (BNP) also showed significant differences between groups. Other baseline characteristics are presented in Table 1.

**Table 1.** Baseline characteristics of the patients by onset time of AKI.

	Overall (*n* = 858)	Non–AKI (*n* = 588)	AKI at admssion (*n* = 226)	Hospital-acquired AKI (*n* = 44)	*p*
Age (years)	55.6 ± 20.2	49.9 ± 18.6	59.7 ± 19.4^‡^	75.2 ± 18.0^‡^	<0.001
Male (%)	37.1	35.9	40.7	34.1	0.41
Vital signs					
Systolic BP (mmHg)	131.3 ± 20.3	131.1 ± 19.4	129.5 ± 21.3	142.3 ± 23.9^‡^	0.001
Diastolic BP (mmHg)	82.4 ± 13.1	83.5 ± 13.0	79.6 ± 13.3^‡^	82.2 ± 11.9	0.001
Mean BP (mmHg)	99.8 ± 13.0	99.0 ± 14.1	96.0 ± 13.9^†^	102.2 ± 14.1	0.004
Body temperature (°C)	36.9 ± 0.7	36.9 ± 0.6	37.0 ± 0.8	36.7 ± 0.5	0.10
Heart rate (beats per min)	86.3 ± 15.5	86.1 ± 15.0	87.6 ± 16.6	82.7 ± 16.0	0.13
Respiratory symptoms (%)	52.8	52.6	57.5	31.8^†^	0.01
Diabetes (%)	16.5	13.3	20.4^‡^	38.6^‡^	<0.001
Hypertension (%)	33.6	27.3	43.1^‡^	68.2^‡^	<0.001
CKD (%)	4.3	2.6	5.8	20.5^‡^	<0.001
AKI stage (%)					<0.001
Stage 1	69.6	–	71.7	59.1	
Stage 2	20.4	–	20.8	18.2	
Stage 3	10.0	–	7.5	22.7	
ICU admission (%)	4.7	0.3	10.6	31.8	<0.001
Laboratory findings					
Hemoglobin (g/dl)	13.6 ± 2.1	13.8 ± 1.7	13.1 ± 2.1^†^	12.3 ± 1.9^‡^	<0.001
WBC (×10^3^/µl)	5.9 ± 2.8	5.3 (4.2–6.8)	5.3 (4.1–7.1)	6.3 (4.6–10.3)	0.32
Platelet (×10^3^/mL)	182.0 (144.0–231.5)	188.0 (151.0–237.0)	157.5 (128.7–202.0)^‡^	174.0 (139.8–214.0)^†^	<0.001
Albumin (g/dL)	4.2 ± 0.5	4.4 ± 0.5	4.1 ± 0.6^†^	3.7 ± 0.6^‡^	<0.001
Total bilirubin (mg/dL)	0.5 (0.4–0.7)	0.5 (0.4–0.7)	0.5 (0.4–0.7)	0.4 (0.3–0.7)	0.66
CRP (mg/dL)	0.7 (0.1–3.0)	0.6 (0.1–2.4)	2.3 (0.7–5.4)^‡^	2.9 (0.4–7.1)^‡^	<0.001
BNP (pg/mL)	14.0 (6.0–36.9)	9.9 (3.1–25.1)	14.8 (5.1–44.2)^‡^	69.8 (27.5–134.2)^‡^	<0.001
Ct value	20.3 (16.0–26.5)	19.8 (15.9–26.0)	20.1 (16.4–26.3)	19.1 (14.8–26.3)	0.47

^†^*p* < 0.01; ^‡^*p* < 0.001 compared with the non-AKI group.

AKI: acute kidney injury; BP: blood pressure; CKD: chronic kidney disease; WBC: white blood cell; CRP: C-reactive protein; BNP: brain natriuretic peptide; Ct: cycle threshold.

### Timing of AKI and mortality outcome

**Figure 2.**
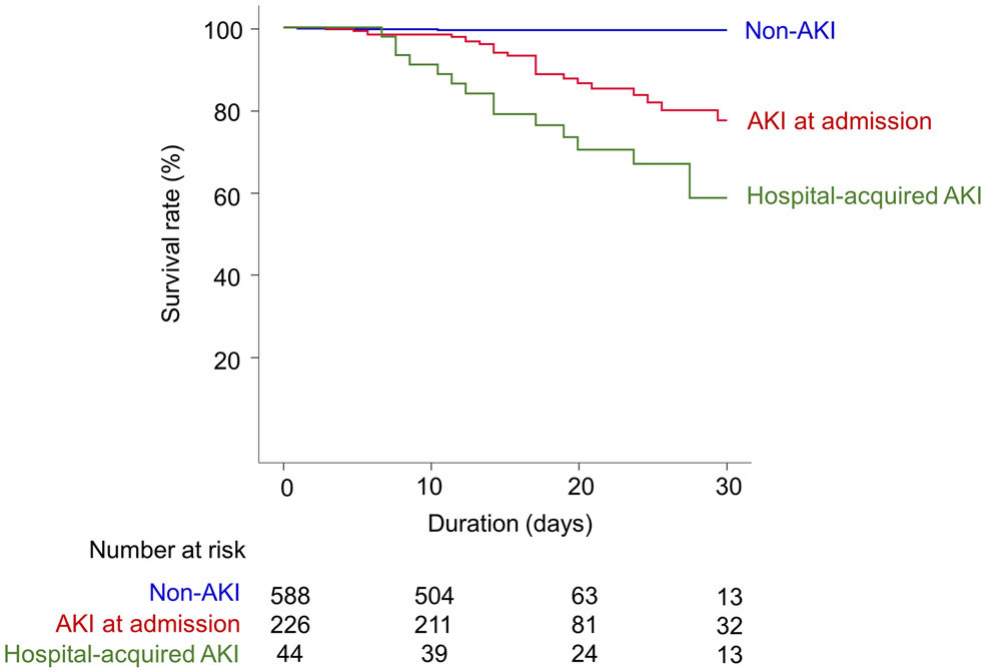
Kaplan–Meier survival curves according to the onset time of AKI.

Among a total of 858 patients, 50 patients (5.8%) died. The mortality incidence was 3.8 deaths per 1,000 person-days. In the Kaplan–Meier survival curves, the survival rates differed according to the timing of AKI (long-rank *p* < 0.001, Figure 2). In the Cox proportional hazards regression, the development of AKI was independently associated with mortality outcome after adjustment for multiple variables, and the hazard for mortality was higher in the hospital-acquired AKI group than in the AKI at admission group (Table 2).

**Table 2.** Risks of mortality outcomes according to onset time of AKI.

Variables	Unadjusted HR (95% CI)	*p*	^†^Adjusted HR (95% CI)	*p*
Non AKI	1 (Reference)		1 (Reference)	
AKI at admission	9.96 (3.457–28.674)	<0.001	9.87 (2.812–34.673)	<0.001
Hospital-acquired AKI	17.46 (5.630–54.147)	<0.001	13.74 (3.572–52.844)	<0.001

^†^Adjusted for age, sex, hypertension, diabetes, chronic kidney disease, hemoglobin, albumin, CRP, and BNP.

HR: hazard ratio; AKI: acute kidney injury.

### Patient characteristics by recovery from AKI at admission

Of 226 patients with AKI at admission, 104 (46.0%) recovered within 48 h, 83 (36.7%) had AKI beyond 48 h and recovered in 7 days, and 39 (17.3%) showed no recovery from AKI on Day 7. No recurrence of AKI was observed within the first 7 days. Patients in the delayed recovery or persistent AKI group were more likely to be older and male and have underlying CKD than those in the early recovery group. Laboratory findings such as hemoglobin, platelet count, serum albumin, CRP and BNP also showed significant differences between groups. Other baseline characteristics are presented in Table 3.

**Table 3.** Baseline characteristics of the patients by recovery of AKI at admission.

Variables	Early recovery (*n* = 104)	Delayed recovery (*n* = 83)	Persistent AKI (*n* = 39)	*p*
Age (years)	54.5 ± 19.2	61.2 ± 18.6	70.5 ± 16.6^†^	0.01
Male (%)	33.7	44.6	51.3	0.11
Vital signs				
Systolic BP (mmHg)	125.6 ± 19.0	134.2 ± 20.8	129.7 ± 25.9	0.08
Diastolic BP (mmHg)	79.4 ± 11.9	80.7 ± 15.1	78.1 ± 12.7	0.12
Mean BP (mmHg)	94.3 ± 12.9	98.6 ± 14.3	95.3 ± 15.2	0.13
Body temperature (°C)	37.2 ± 0.8	37.0 ± 0.8	36.9 ± 0.8	0.42
Heart rate (beats per min)	88.5 ± 17.6	88.5 ± 14.6	80.6 ± 16.1*	0.03
Respiratory symptoms (%)	61.5	55.4	51.3	0.48
Diabetes (%)	19.2	16.9	30.8	0.19
Hypertension (%)	35.6	47.6	53.8	0.09
CKD (%)	1.0	6.0^‡^	17.9^‡^	0.001
AKI stage (%)				<0.001
Stage 1	85.6	72.3	33.3^‡^	
Stage 2	13.4	20.5	41.0^‡^	
Stage 3	1.0	7.2	25.7^‡^	
Laboratory findings				
Hemoglobin (g/dl)	13.5 ± 2.1	12.9 ± 2.2	12.5 ± 2.3*	0.01
WBC (×10^3^/µl)	5.3 (4.1–6.7)	5.4 (4.0–7.6)	5.5 (4.5–7.8)	0.33
Platelet (× 10^3^/mL)	170.0 (137.3–201.0)	157.0 (132.0–203.0)	138.0 (105.0–200.0)	0.14
Albumin (g/dL)	4.2 ± 0.5	4.0 ± 0.7	3.8 ± 0.7^†^	0.01
Total bilirubin (mg/dL)	0.5 (0.4–0.7)	0.5 (0.4–0.7)	0.5 (0.4–0.7)	0.68
CRP (mg/dL)	2.2 (0.9–4.8)	2.3 (0.3–5.7)	2.2 (0.9–7.0)	0.02
BNP (pg/mL)	9.3 (3.1–27.4)	19.9 (7.5–59.8)^‡^	35.6 (12.9–124.5)^‡^	<0.001
Ct value	19.8 (16.0–26.0)	18.9 (15.8–23.9)	19.4 (13.7–24.6)	0.30

*p < 0.05; ^†^*p* < 0.01; ^‡^*p* < 0.001 compared with the early recovery group.

AKI: acute kidney injury; BP: blood pressure; CKD: chronic kidney disease; WBC: white blood cell; CRP: C-reactive protein; BNP: brain natriuretic peptide; Ct: cycle threshold.

### Recovery from AKI at admission and mortality outcome

**Figure 3.**
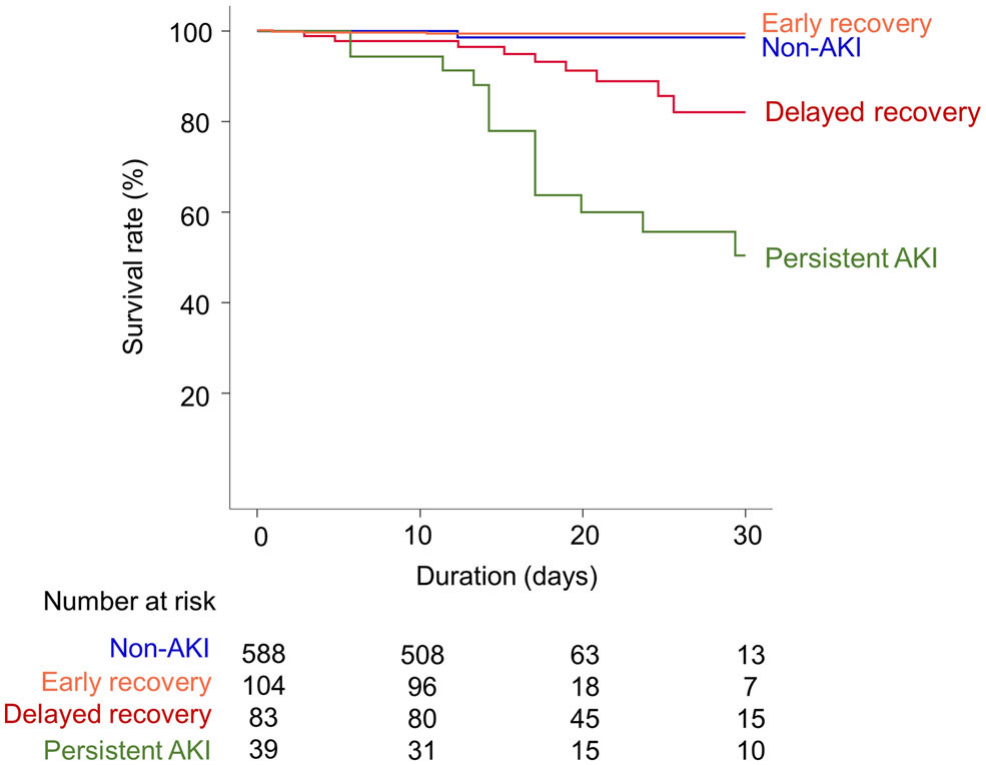
Kaplan–Meier survival curves according to recovery from AKI at admission.

Figure 3 shows the Kaplan–Meier survival curves according to recovery from AKI at admission. There was no significant difference in the survival curves between the patients in the early recovery and non-AKI groups (*p* = 0.852). However, the delayed recovery group and the persistent AKI group had significantly higher mortality than the non-AKI group (*p* = 0.005). In the Cox proportional hazards regression model, delayed recovery or persistent AKI significantly increased the risk of hospital mortality (Table 4).

**Table 4.** Hazard ratios for hospital mortality according to recovery from AKI at admission.

			Model 1^†^		Model 2^‡^	
Variables	Unadjusted HR (95% CI)	*p*	Adjusted HR (95% CI)	*P*	Adjusted HR (95% CI)	*p*
Non–AKI	1 (Reference)		1 (Reference)		1 (Reference)	
Early recovery	1.16 (0.129–10.435)	0.893	1.16 (0.121–11.186)	0.897	0.99 (0.109–8.946)	0.992
Delayed recovery	6.14 (1.810–20.827)	0.004	5.00 (1.272–19.633)	0.021	3.38 (1.105–13.603)	0.034
Persistent AKI	15.65 (5.014–48.818)	<0.001	17.29 (4.989–59.906)	<0.001	16.66 (5.383–51.590)	<0.001

^†^Adjusted for age, sex, hypertension, diabetes, and chronic kidney disease.

^‡^Adjusted for age, sex, hypertension, diabetes, chronic kidney disease, hemoglobin, albumin, CRP, and BNP.

### Predictive factors for delayed recovery or persistent AKI

Table 5 shows a logistic regression analysis to identify the predictive factors for either delayed recovery or persistent AKI. Old age, low hemoglobin level, and low platelet count were potential predictive factors of a longer duration of AKI.

**Table 5.** Odds ratios for delayed recovery or persistent AKI.

Variables	Unadjusted OR (95% CI)	*p*	Adjusted OR (95% CI)	*p*
Age (per 10 year increase)	1.46 (1.310–1.623)	<0.001	1.41 (1.203–1.611)	0.001
Male (vs. female)	1.59 (1.078–2.344)	0.02	1.54 (0.981–2.423)	0.06
Diabetes (vs. no diabetes)	1.64 (1.009–2.652)	0.05	0.75 (0.419–1.353)	0.34
Hypertension (vs. no hypertension)	2.46 (1.662–3.646)	<0.001	1.01 (0.596–1.730)	0.95
CKD (vs. no CKD)	4.61 (2.123–10.006)	<0.001	2.11 (0.849–5.261)	0.11
Hemoglobin (per 1 g/dl increase)	0.77 (0.698–0.852)	<0.001	0.84 (0.740–0.964)	0.01
Platelet (per 10^4^/mL increase)	0.93 (0.903–0.964)	<0.001	0.99 (0.991–0.997)	<0.001
Albumin (per 1 g/dL increase)	0.33 (0.230–0.465)	<0.001	0.74 (0.451–1.222)	0.24
Total bilirubin (per 1 mg/dL increase)	0.78 (0.795–1.551)	0.48		
CRP (per 1 mg/dL increase)	1.09 (1.055–1.140)	<0.001	1.05 (0.997–1.095)	0.07
BNP (per 10 pg/mL increase)	1.01 (0.997–1.018)	0.15		

OR: odds ratio; CKD: chronic kidney disease; CRP: C-reactive protein; BNP: brain natriuretic peptide.

## Discussion

The present study assessed the trajectory of AKI as a predictive factor for mortality in hospitalized patients with COVID-19. The occurrence of AKI increased the risk of mortality regardless of the time of onset; however, hospital-acquired AKI conferred a higher risk than AKI at admission. Importantly, the duration of AKI at admission was also significantly associated with the risk of mortality.

The incidence of AKI has been reported by previous studies, ranging from 0.5 to 80% among hospitalized patients with COVID-19 [18], and these discrepancies in the reported incidence likely reflect differences in sample sizes and cohort characteristics according to patterns of identifying AKI or the disease severity of COVID-19. Few studies have explored the temporal relationship between SARS-CoV-2 infection and the development of AKI. Some studies reported that AKI developed throughout the hospital stay, at an average of 5 to 9 days after admission [19,20], and other studies reported AKI at hospital admission, with an incidence ranging from 1 to 29% [21,22]. Our data reported that one in four COVID-19 patients presented with AKI at admission, and an additional 6% developed AKI throughout hospitalization. Given that the time from confirmation of COVID-19 to hospitalization was less than a day in this cohort, we found that a considerable proportion of patients developed early AKI after infection. As in many previous studies, although discrepancies exist in the timing of identifying AKI, we found that early AKI after COVID-19 infection is associated with poor short-term survival in COVID-19 patients [23–25].

Additionally, our data demonstrate differences in demographics, comorbidity profiles, and survival outcomes according to the onset time of AKI. Compared with the AKI at admission group, the hospital-acquired AKI group had significantly higher levels of hs-CRP, which represents a systemic inflammatory response, higher levels of BNP, disproportionately severe AKI, and higher mortality. This result was congruent with a previous retrospective study that defined early AKI as AKI onset occurring before the onset date of any other organ dysfunction and late AKI as all other circumstances [26]. In our study, it could be assumed that the difference in mortality rate was attributed to the cause of AKI; AKI at admission was predominantly caused by dehydration or direct viral infection of the kidney, and hospital-acquired AKI occurred with secondary infection or multiorgan failure following acute respiratory distress syndrome (ARDS).

Renal recovery was significantly associated with a low risk of mortality in both non-COVID-19 and COVID-19 patients [13,27–30]. On the other hand, the development of AKI increases the risk of long-term mortality even in the presence of renal recovery [16]. Our finding that persistent AKI at 7 days after admission was associated with a high risk of hospital mortality is consistent with a previous prospective multicenter cohort study [25]; however, we added an analysis by duration of AKI. The duration of AKI intersects with the period of renal recovery. Patients experiencing transient AKI generally exhibit prompt renal recovery, whereas those with prolonged AKI are considered to have a delayed renal function restoration. The Acute Disease Quality Initiative Workgroup, in a consensus report, has defined transient AKI as lasting less than 48 h, while persistent AKI is characterized by a duration that extends beyond 48 h. Furthermore, they have introduced a new term to categorize AKI cases that persist between 7 to 90 days, which is referred to as acute kidney disease [31]. A meta-analysis of 19 global studies has revealed that the duration of AKI significantly impacts long-term mortality, cardiovascular outcomes, and the development of CKD [32]. In the present study, despite the brief follow-up period, it appears that the duration of early AKI provides information on the risk of mortality in patients with COVID-19. Additionally, our results indicate a significant association of old age and anemia with increased adjusted odds of persistent AKI. This suggests that AKI may partially contribute to the elevated mortality risk previously observed among these subgroups in the context of COVID-19 [33–36].

This study provides valuable information, however, it is important to acknowledge the existence of several limitations that warrant attention. The retrospective design of the study may have introduced unmeasured biases and confounders that could have influenced the results of our analysis. Furthermore, the potential impact of differences in practice on mortality outcomes was not evaluated in this study. A history of other diseases or the use of medications, such as treatments for COVID-19, were not included. The cause of death could not be ascertained in the present dataset. Long-term outcomes after discharge according to the trajectory of hospital-acquired AKI were not available in this cohort. Future research on this issue will provide us with more evidence for the risk stratification of COVID-19 survivors.

## Conclusions

The occurrence of AKI in patients hospitalized with COVID-19 is prevalent and has a significant correlation with short-term mortality. The progression of early AKI offers significant information for evaluating short-term patient outcomes. Hence, it is recommended to closely monitor renal function shortly after infection in order to classify the risk and effectively prevent the adverse outcomes in COVID-19 patients.

## Ethical approval

This study was approved by the institutional review board of Seoul National University Boramae Medical Center (no. 20-2021-88) and complied with the Declaration of Helsinki. The requirement for informed consent was waived by the review board.

## Data Availability

The data are available from the authors upon request.
